# Financial Toxicity Among Cancer Patients in Slovenia

**DOI:** 10.1002/cam4.70891

**Published:** 2025-04-21

**Authors:** Marjeta Skubic, Katja Vöröš, Mojca Bavdaž, Petra Došenović Bonča, Andraž Perhavec, Tjaša Redek, Ivica Ratoša, Helena Barbara Zobec Logar

**Affiliations:** ^1^ Faculty of Medicine University of Ljubljana Ljubljana Slovenia; ^2^ School of Economics and Business University of Ljubljana Ljubljana Slovenia; ^3^ Division of Surgical Oncology Institute of Oncology Ljubljana Ljubljana Slovenia; ^4^ Division of Radiotherapy Institute of Oncology Ljubljana Ljubljana Slovenia

**Keywords:** cancer burden, cancer patients, COST‐FACIT, EORTC QLQ‐C30, financial burden, financial toxicity, quality of life

## Abstract

**Background:**

The ageing population, increasing medical costs and a number of newly diagnosed cancer cases among the working population are increasing the financial burden on healthcare systems. The extent of financial toxicity in Slovenian patients has been insufficiently researched, as has its impact on quality of life (QoL).

**Methods:**

To evaluate financial toxicity, the Functional Assessment of Chronic Illness Therapy (COST‐FACIT) questionnaire was translated into Slovenian, validated, introduced and compared with the European Organisation for Research and Treatment of Cancer Core Quality of Life Questionnaire (EORTC QLQ‐C30). Additional questions were incorporated into the questionnaire to further quantify and objectify financial toxicity.

The study was cross‐sectional. The statistical analysis was based on descriptive and inferential statistics and exploratory data analysis.

**Results:**

Out of 590 analysed participants, financial toxicity was absent in 57.2% but present at mild to moderate levels in 42.8%. Key risk factors included lower income, age ≤ 65, employment, active oncologic treatment, rural residence and religious affiliation. Post hoc analyses showed higher financial toxicity in those with ≤ 600 EUR monthly income, employed patients and spiritual individuals, while cancer type showed no significant differences. The correlation between financial toxicity and QoL was mild. Objective measures of financial toxicity include direct costs (e.g., transportation, supplements and medical devices) and indirect costs (e.g., loss of income) associated with disease and treatment, which burdened more than 40% of the studied population.

**Conclusions:**

The COST‐FACIT proved to be a helpful screening tool for identifying patients at risk, even in a public healthcare system such as the Slovenian system. On average, financial toxicity is low due to the publicly funded financial system covering the treatment and rehabilitation of malignant diseases. The age structure of cancer patients and secure pension income further contribute to this outcome.

## 1 Background

In the literature, financial toxicity refers to the objective and subjective financial burdens to which cancer patients and their families are exposed due to the disease and its treatment, encompassing all direct and indirect costs [1]. Financial toxicity is known to have a negative impact on a range of clinically important disease outcomes, including treatment adherence, quality of life (QoL), disease symptoms and survival [1, 2, 3, 4]. Patients facing financial strain may be forced to delay or forgo necessary treatments, worsening their prognosis.

Cancer is one of the leading causes of morbidity and mortality worldwide, and Slovenia is no exception. GDP per capita in purchasing power parity reached 92% of the EU average in 2023 and ranks 14th in the 2023 ranking [5]. With a population of just over 2 million, the country is experiencing a demographic shift toward an ageing population, with individuals aged 65+ increasing from 10.6% in 1990 to 21.4% in 2023 [6, 7, 8, 9]. This shift is driving a rapid rise in cancer cases, with newly diagnosed cases increasing from 13,700 in 2013 to over 17,000 in 2021 [10]. While cancer incidence is highest among the elderly, a substantial portion (35%) of newly diagnosed cases occur in the working‐age population (20–65 years) [10, 11]. Given this trend, the economic burden of cancer on individuals, families and the healthcare system is expected to grow significantly.

Slovenia has a public healthcare system based on the Bismarck model, which is financed jointly by employers and employees through payroll deduction. Compulsory employment‐based social health insurance (CHI) is provided by a single public insurer, the Health Insurance Institute of Slovenia (HIIS) and covers more than 99% of permanent residents [12]. CHI provides coverage for a wide range of primary, secondary and tertiary level healthcare services as well as medicines and medical devices, compensation for prolonged sick leave, and, in some cases, travel expenses to health facilities. Until the end of 2023, a wide array of services was not fully covered by CHI, with co‐payments ranging from 10% to 90% of the fees set by HIIS, depending on their complexity and necessity. Health services for children, maternal care, disease prevention and early detection, organ and tissue transplantation, emergency health care services and treatment and rehabilitation of malignant diseases received full coverage by CHI [13]. Most co‐payments, however, were not covered by patients' out‐of‐pocket expenses but through complementary voluntary health insurance for the full coverage of co‐payments (CoVHI) that covered about 95% of the population eligible for CHI. Furthermore, patients receiving social benefit payments were exempt from paying co‐payments. While co‐payments varied over a wide range, their average was not very high. This is indicated by the share of CoVHI in overall current health expenditures, which amounted to 13.4% in 2023. Given the wide range of services covered by CHI and legislative changes in 2024 that abolished co‐payments and introduced full coverage of all CHI services, the health system is struggling to keep up with growing needs, resulting in extended waiting times, a significant drawback of the system. While waiting times are also contributing to the growth of supplementary voluntary insurance, covering faster access to services, greater choice of health care providers, or enhanced amenities, premiums for such insurance products remained below 5% of all voluntary insurance premiums in 2023, given the pivotal role of CoVHI [12, 13].

While publicly funded healthcare systems include safeguards against financial toxicity, studies suggest that 22%–27% of cancer patients in these systems still experience financial distress [14, 15, 16]. For instance, financial toxicity has been documented in European countries with similar public healthcare models, such as Italy, Germany, the Netherlands and Ireland [17, 18, 19, 20, 21, 22]. In contrast, financial toxicity in the United States, where healthcare is predominantly privatised, affects 39%–64% of cancer patients, often leading to treatment non‐adherence, financial distress and health inequities [23, 24, 25, 26]. Furthermore, studies suggest that financial burden as a result of cancer treatment and care is one of the strongest predictors of poor quality of life in cancer survivors [27, 28]. Despite the presence of public health coverage, financial barriers persist in many countries due to hidden costs such as out‐of‐pocket expenses for non‐covered medications, transportation, lost wages and home care services and are major causes of inequity in access to health care [16]. However, no such data exist for Slovenia.

Given these gaps, this study aimed to assess the prevalence of financial toxicity among Slovenian cancer patients and establish a baseline before recent health insurance reforms. To achieve this, we translated and validated the COST‐FACIT‐v2 questionnaire into Slovenian and examined its psychometric properties. Furthermore, we assessed financial toxicity's relationship with quality of life (QoL) and financial difficulties (FI) by comparing COST‐FACIT‐v2 results with the European Organisation for Research and Treatment of Cancer Core Quality of Life Questionnaire (EORTC QLQ‐C30).

The primary objective of this study was twofold: first, to validate the Slovenian translation of the Comprehensive Score for Financial Toxicity (COST‐FACIT‐v2) instrument among adult cancer patients and second, to assess the prevalence and predictors of financial toxicity within a representative oncology population in Slovenia. Specifically, the study aimed to identify potential risk categories based on financial toxicity levels and to evaluate their association with overall quality of life (QoL). It was hypothesised that higher financial toxicity, as measured by the COST‐FACIT‐v2, would be positively correlated with self‐reported financial difficulties and negatively correlated with overall QoL, as assessed by the Global Health Status/QoL (GHS/QoL) domain of the EORTC QLQ‐C30 questionnaire. To complement the subjective assessment of financial distress, the survey included additional items addressing objective indicators of financial burden, such as out‐of‐pocket treatment costs and other healthcare‐related expenditures (see Data S1, Section C).

By studying financial toxicity in Slovenia, we aimed to highlight hidden financial burdens and provide insights into patient‐centred interventions or policy adjustments that could improve cancer care and financial protection. Additionally, comparing Slovenia's findings with those from countries with different healthcare models provided valuable insights into the strengths and weaknesses of public healthcare systems in mitigating financial toxicity.

## 2 Methods

### 2.1 Study Design, Sample Size and Participant Recruitment

The cross‐sectional study was conducted between June and October 2023. Participants were recruited at the Institute of Oncology Ljubljana (The Institute of Oncology Ljubljana is Slovenia's leading comprehensive cancer center, delivering care to the majority of cancer patients in the country. Notably, it serves as the central radiotherapy center, offering both external beam and brachytherapy treatments, making it the primary hub for multimodal cancer care.), the largest cancer treatment centre in Slovenia, serving approximately 70% of the national cancer patient population [29].

#### 2.1.1 Inclusion and Exclusion Criteria

Inclusion criteria were: adults (≥ 18 years) with a confirmed diagnosis of cancer, regardless of cancer type, treatment status, or stage, able to understand Slovenian and provide informed consent.

Exclusion criteria included: patients with significant cognitive impairment or severe mental illness limiting their ability to complete the questionnaire, and patients in end‐of‐life or hospice care.

The required sample size was calculated using G*Power version 3.1 software with the following parameters: significance level (*α*) 0.05, statistical power (1*‐β*) 0.95 (95% power, providing high confidence in detecting an actual effect) and effect size (*f*) 0.25. The sample size was calculated for net household income, sufficient for comparing all other variables with fewer groups. Based on these assumptions, the estimated required sample size per group was 324 participants. To ensure robustness in the analysis and account for potential data loss or missing responses, the study aimed to recruit a higher number of participants, providing a more precise estimate of financial toxicity levels. According to Comrey and Lee, a sample size of at least 500 subjects could be considered very good for performing factor analysis [30]. At the same time, the initial recruitment strategy employed convenience sampling; purposive and quota sampling elements were subsequently incorporated to enhance sample representativeness. Specifically, as data collection progressed, we monitored the distribution of cancer types within the sample to roughly approximate the national cancer incidence. Furthermore, efforts were made to ensure heterogeneity across key patient characteristics, such as age and gender.

### 2.2 Questionnaire Translation and Validation Process

To validate the Slovenian translation of the COST‐FACIT questionnaire for clinical use, we followed FACIT.org's methodology which requires: two independent forward translations, from English to Slovenian by two translators working independently from one another, reconciliation by a third translator, back translation performed by a fourth translator, review/finalisation by a fifth translator, proofreading and cognitive testing with 10 patients who complete the test version of the questionnaire and then answer questions from a cognitive debriefing script prepared by FACIT.org [30]. Following this methodology, FACIT.org approved using the validated COST‐FACIT questionnaire in the Slovenian context. The EORTC QLQ‐C30 version 3.0 had already been validated in Slovenia.

### 2.3 Data Collection and Measurement Instruments

Data collection involved a three‐part patient questionnaire and documentation review. The questionnaire comprised the EORTC QLQ‐C30, the COST‐FACIT and questions on background information about sociodemographics, socioeconomic status, disease characteristics and treatment history (Data S1). It also included questions designed to quantify the direct and indirect financial burden on patients. These background questions were developed collaboratively by the research team, leveraging expertise in oncology, economics and questionnaire design. The questionnaire was designed for self‐administration. Most patients completed the questionnaire in inpatient wards before outpatient radiotherapy, in the day hospital, or during follow‐up visits. Trained researchers were frequently available to assist as needed. A subset of patients chose to complete the questionnaire online at their convenience.

The COST‐FACIT‐v2 assesses subjective financial distress in cancer patients through a 12‐item questionnaire rated on a 0–4 Likert scale. The final score is calculated via the first 11 items, with scores ranging from 0 to 44. Lower scores indicate greater financial toxicity and are classified into four grades (G0–G3) [31].

The EORTC QLQ‐C30 version 3.0 consists of 30 items. The first five items assess overall QoL, whereas the remaining items evaluate QoL in the past week on scales of 1–4 and 1–7. The questionnaire includes multi‐item scales and single‐item measures covering functional and symptom scales, with scores ranging from 0 to 100. The Financial Difficulties (FI) item corresponds to question 28 of the EORTC QLQ‐C30, while the GHS/QoL pertains to questions 29 and 30. In this questionnaire, higher scores indicate better functioning or more severe symptoms (e.g., financial difficulties), depending on the scale. A 10‐point difference is typically regarded as clinically significant. Further information on scoring is available in the EORTC scoring manual [32].

### 2.4 Statistical Analysis Methods

The distribution of the variables was assessed for normality via the Kolmogorov–Smirnov and Shapiro–Wilk tests. As most variables were non‐normally distributed, a nonparametric Kruskal–Wallis test was used to compare the median values. For variables with more than two categories, pairwise comparisons were conducted using the Mann–Whitney *U* test. To control for multiple comparisons, a Bonferroni correction was applied, adjusting the significance threshold based on the number of groups within each variable. For cancer types (5 groups), the adjusted significance level was set at *p* ≤ 0.005, while for religious affiliation and employment status (3 groups), it was set at *p* ≤ 0.0167. The same approach was applied to other categorical variables with multiple groups. Pearson's chi‐square test was used to assess associations between categorical variables. The reliability of the COST‐FACIT questionnaire was measured with Cronbach's alpha. Spearman's rank correlation coefficient (*ρ*) was used to evaluate the associations between the COST‐FACIT and the EORTC QLQ‐C30. Statistical analysis was performed with IBM SPSS version 29. Differences were considered significant at *p* ≤ 0.05 (two‐tailed).

### 2.5 Ethical Considerations

The study was approved by the Ethics Committee of the Institute of Oncology Ljubljana on 16 March 2023, the Commission for the Peer Review of Research Protocols of the Institute of Oncology Ljubljana on 2 March 2023, the Expert Council of the Institute of Oncology Ljubljana on 23 May 2023 (number ERIDNPVO‐0031/2023) and the Commission for Medical Ethics of the Republic of Slovenia on 18 April 2023 (number 0120–105/2023/3). The study was conducted in accordance with the ethical principles of the Declaration of Helsinki. Before participating in the study, all the participants provided informed consent for the processing of their personal data.

## 3 Results

### 3.1 Participants' Characteristics

**FIGURE 1:**
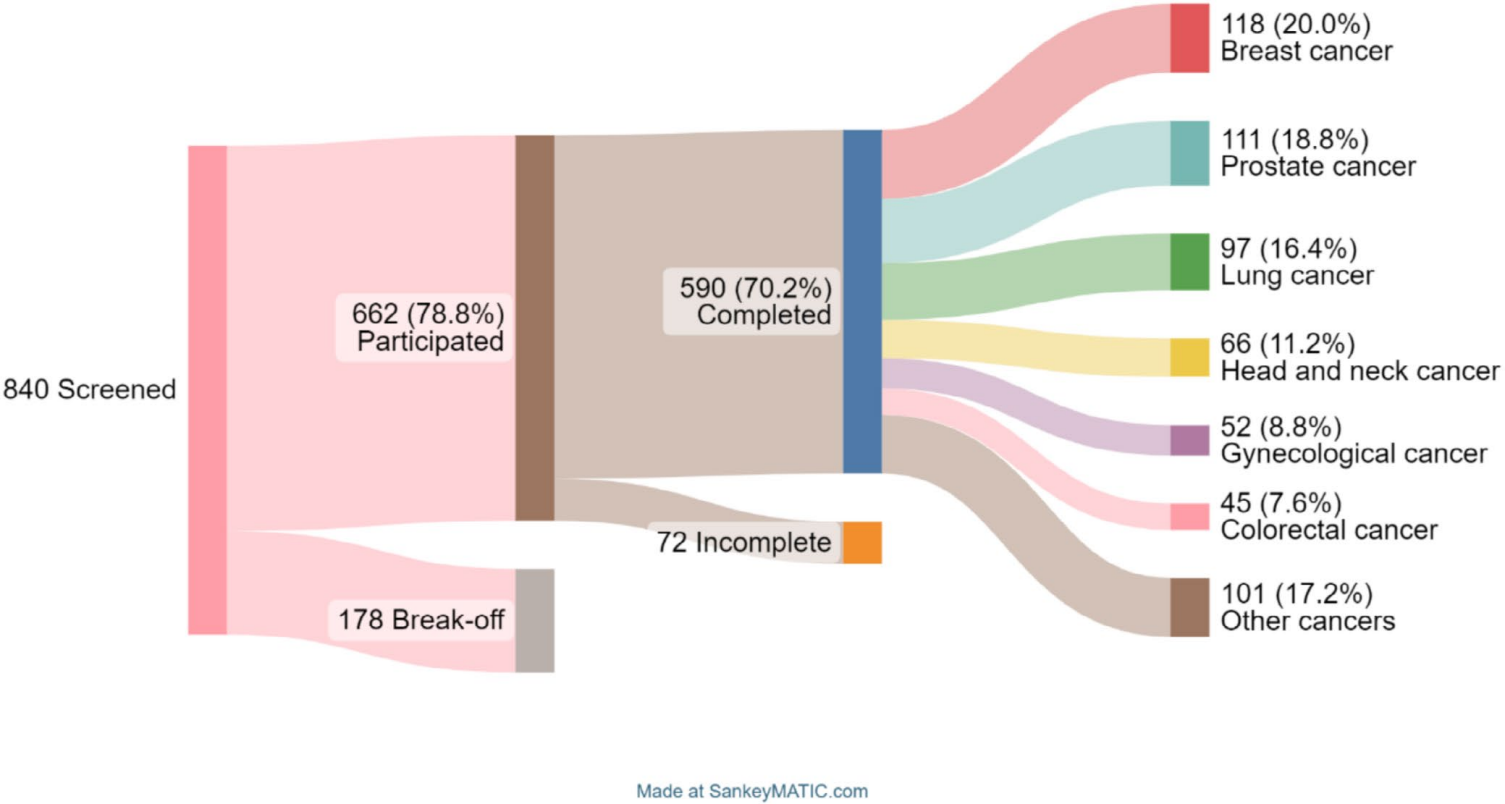
Recruitment of patients and proportions by diagnosis. Other types of cancer were rarer primary tumours, such as leukaemia, sarcoma, pancreatic cancer, liver cancer and anaplastic ependymomas of the spinal cord and head.

Among the 840 screened patients, 590 completed the questionnaires. Figure 1 outlines the recruitment, attrition and final selection process, as well as the most common primary diagnoses.

The average age of the respondents was 62.7 ± 12.9 years. The average duration of cancer survivorship was 33.4 ± 43.9 months. During this period, 49.5% of patients (*n* = 292) underwent surgery, 74.6% (*n* = 440) received radiation therapy and 25.3% (*n* = 149) underwent hormonal treatment. Additionally, 52.2% (*n* = 308) received chemotherapy, 14.7% (*n* = 87) underwent targeted or immunotherapy treatment and 0.5% (*n* = 3) received other forms of treatment. Patients were divided into two age groups based on expected retirement age. The sample was balanced in terms of gender, age, living environment and employment status. Most (93.4%) had CHI and CoVHI for the full coverage of co‐payments. The average sickness‐related absence from work was 11.9 months (range: 1–72 months). The detailed characteristics of the study sample can be found in Table 1.

**TABLE 1:** Characteristics of the study sample with demographic and clinical characteristics of participants, including financial and social support variables.

Group	Number of patients	Proportion
	*N*	%
Sociodemographic characteristics		
Age groups (years)		
≤ 65 years	326	55.3
> 65 years	264	44.7
Sex		
Male	294	49.8
Female	293	49.7
Missing	3	0.5
Educational level		
(Incomplete) primary	83	14.1
Secondary	321	54.4
Postsecondary or above	181	30.7
Missing	5	0.8
Religion		
Atheist	409	69.3
Religious	119	21.9
Other	15	2.5
Missing	37	6.3
Living environment		
Rural	298	50.5
Urban	286	48.5
Socioeconomic characteristics		
Living alone		
No	508	86.2
Yes	82	13.9
Type of health insurance (*N* = 589)		
Only CHI	17	2.9
CoVHI (in addition to CHI)	534	90.5
Supplementary (in addition to CHI and CoVHI)	36	6.1
Other^a^	2	0.3
Employment status		
Employed	258	43.7
Unemployed	22	3.7
Retired	303	51.4
Student^b^	1	0.2
Other^c^	4	0.7
Missing	2	0.3
Net household income per person per month (euro)		
≤ 300	18	3.1
Above 300 up to 600	105	17.8
Above 600 up to 900	208	35.3
Above 900 up to 1200	109	18.5
Above 1200 up to 1500	81	13.7
> 1500	65	11.0
Missing	4	0.7
Disease and treatment		
Site of the primary tumour		
Breast cancer	118	20.0
Prostate cancer	111	18.8
Lung cancer	97	16.4
Head and neck cancer	66	11.2
Other	198	33.6
Recurrence		
No	496	84.1%
Yes	94	15.9%
Active treatment		
No	181	30.7%
Yes	408	69.1%
Missing	1	0.2%
Treatment type		
Unimodal	166	28.1%
Multimodal	423	71.7%
Missing	1	0.2%

^a^ One person insured through the protection of displaced persons and refugees, one person who worked and was insured abroad.

^b^ Only one patient was a student, and it was omitted from the analysis.

^c^ One patient was on sick leave, one was working through a service contract, one had a widow's pension, and two were family assistants.

### 3.2 COST‐FACIT and EORTC QLQ‐C30

**FIGURE 2:**
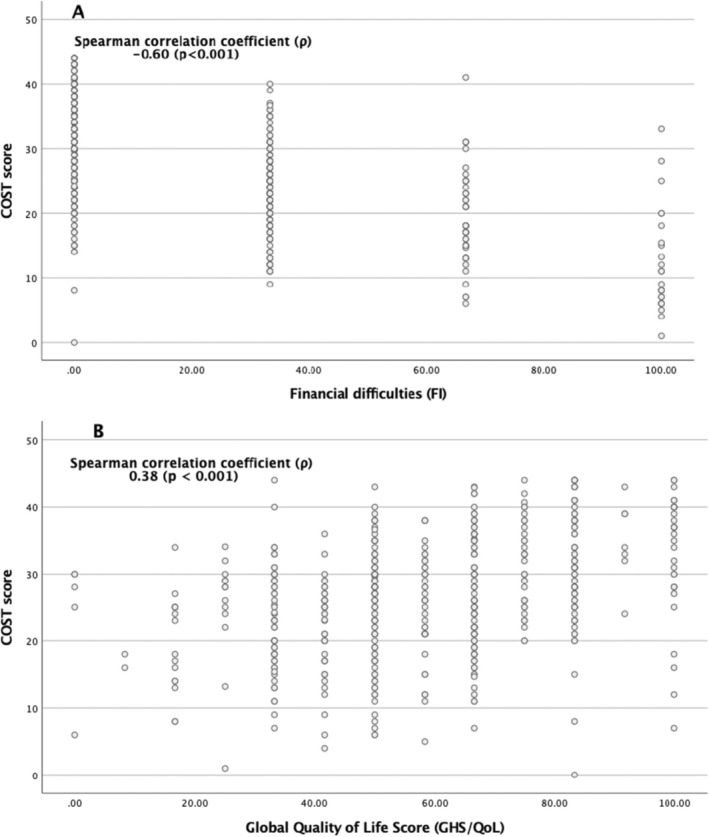
Correlation between COST‐FACIT score and (A) EORTC‐QLQC30 financial difficulties (FI) (B) and quality of life (QL2) scale.

The COST‐FACIT questionnaire mean score was 26.7. Grade G0 was found in 53.7%, G1 in 33.1%, G2 in 7.3% and G3 in 0.2% of the samples. Scores were not calculated for 5.8% of the participants because they did not respond to at least two questions. The Cronbach's alpha was 0.781, indicating acceptable internal consistency, and the interclass correlation (ICC) was 0.724, with a 95% confidence interval [0.689–0.758], indicating acceptable test–retest reliability. Table 2 compares the COST‐FACIT with the EORTC QLQ‐C30 questionnaire (FI and GHS/QoL measures). The Spearman correlation coefficient (*ρ*) between the COST‐FACIT score and GHS/QoL was 0.38 (*p* < 0.001), indicating a statistically significant mild association. The negative correlation coefficient between the COST‐FACIT score and FI is 0.60 (*p* < 0.001), which indicates a moderate correlation (Figure 2).

**TABLE 2:** Comparison between COST‐FACIT and EORTC QLQ‐C30 FI and GHS/QoL (QL2).

Group	Cost‐FACIT		EORTC QLQ‐C30			
			FI		GHS/QoL (QL2)	
	Mean (SD)	*p*	Mean (SD)	*p*	Mean (SD)	*p*
Age groups (years)						
≤ 65 years	25.1 (9.0)	**< 0.001**	27.0 (30.1)	**< 0.001**	59.2 (21.7)	0.647
> 65 years	28.7 (8.3)		15.5 (24.1)		58.9 (22.2)	
Sex						
Male	27.4 (8.2)	0.115	17.3 (25.5)	**< 0.001**	58.7 (22.0)	0.823
Female	26.0 (9.4)		26.3 (29.9)		59.3 (21.8)	
Educational level						
(Incomplete) primary	24.6 (9.2)	0.070	21.1 (30.4)	0.757	55.1 (25.7)	**0.037**
Secondary	26.8 (8.4)		21.3 (26.7)		61.3 (20.3)	
Postsecondary or above	27.5 (9.4)		23.0 (29.3)		57.1 (22.6)	
Religion						
Atheist	30.1 (7.9)	**< 0.001**	14.0 (23.4)	**0.002**	63.2 (21.0)	0.076
Religious	25.6 (8.9)		24.6 (29.4)		58.0 (21.6)	
Other	26.9 (8.8)		20.0 (24.6)		61.1 (22.9)	
Living environment						
Rural	26.0 (8.9)	**0.032**	20.1 (27.8)	0.066	58.4 (22.1)	0.470
Urban	27.5 (8.8)		20.1 (28.5)		60.1 (21.8)	
Living alone						
No	26.7 (8.7)	0.946	21.4 (27.4)	0.648	59.3 (21.7)	0.456
Yes	26.3 (9.7)		24.7 (31.9)		57.8 (23.1)	
Type of health insurance						
Only CHI	26.7 (8.9)	0.120	21.8 (28.2)	0.774	59.0 (21.9)	0.428
CoVHI (in addition to CHI)	26.8 (8.9)		21.6 (28.0)		58.9 (22.0)	
Supplementary (in addition to CHI and CoVHI)	29.5 (7.2)		18.9 (25.8)		62.5 (20.6)	
Employment status						
Employed	24.5 (9.2)	**< 0.001**	30.1 (30.7)	**< 0.001**	59.0 (21.9)	0.592
Unemployed	26.3 (10.0)		15.8 (28.0)		64.5 (27.8)	
Retired	28.5 (8.0)		15.3 (23.8)		58.5 (21.6)	
Other	32.3 (5.9)		11.1 (19.2)		52.8 (12.8)	
Net household income per person per month (EUR)						
≤ 300	19.7 (9.1)	**< 0.001**	41.2 (40.0)	**< 0.001**	50.5 (29.2)	**0.007**
Above 300 up to 600	21.6 (8.5)		33.3 (30.2)		51.9 (21.9)	
Above 600 up to 900	25.9 (8.2)		21.5 (28.6)		60.2 (20.2)	
Above 900 up to 1200	29.6 (8.7)		17.5 (24.7)		58.9 (21.0)	
Above 1200 up to 1500	29.8 (7.2)		16.2 (22.2)		61.9 (22.5)	
> 1500	30.2 (8.6)		14.5 (24.0)		65.3 (22.0)	
Site of the primary tumour						
Breast cancer	24.9 (9.5)	**0.016**	29.9 (33.0)	**0.001**	63.2 (21.2)	**0.020**
Prostate cancer	28.0 (8.1)		13.8 (21.8)		62.5 (20.2)	
Lung cancer	28.6 (7.6)		17.1 (24.4)		58.8 (20.6)	
Head and neck cancer	27.3 (9.5)		20.3 (30.3)		54.0 (25.9)	
Other	25.8 (9.0)		23.9 (33.3)		56.1 (21.8)	
Recurrence						
No	26.7 (9.0)	0.935	21.9 (28.2)	0.896	59.1 (21.8)	0.982
Yes	26.7 (7.9)		21.2 (27.7)		58.8 (22.3)	
Active treatment						
No	28.7 (8.6)	**0.001**	17.8 (25.2)	**0.039**	65.3 (20.6)	**< 0.001**
Yes	25.8 (8.8)		23.5 (29.0)		56.1 (21.9)	
Treatment type						
Unimodal	27.4 (8.2)	0.247	18.3 (28.3)	**0.020**	58.4 (20.8)	0.598
Multimodal	26.4 (9.1)		23.1 (27.9)		59.5 (22.3)	

*Note:* Statistically significant *p* values are bolded. Clinically significant *p* values for the EORTC QLQ questionnaire, with a difference of more than 10 points, and the FACIT‐COST questionnaire, with a difference of more than 6 points, are bolded and underlined.

Abbreviations: CHI, employment‐based social health insurance; COST‐FACIT, Comprehensive score for Financial Toxicity—Functional Assessment of Chronic Illness Therapy; CoVHI, complementary voluntary health insurance for the full coverage of co‐payments; EORTC QLQC‐30, The European Organisation for Research and Treatment of Cancer Core Quality of Life Questionnaire; FI, Financial difficulties scale; GHS/QoL (QL2), Global Health Status/Quality of Life (Quality of life scale); HIIS, Health Insurance Institute of Slovenia.

Our analysis identified several factors associated with increased financial toxicity among cancer patients. Younger patients experienced higher financial toxicity (*p* < 0.001), as did those with lower net incomes (*p* < 0.001), patients undergoing active treatment (*p* = 0.001) and individuals from rural areas (*p* = 0.032). A Kruskal‐Wallis test revealed significant differences in financial toxicity across various cancer types (*p* = 0.016), indicating that at least one group differed from the others. To further explore pairwise differences, post hoc Mann–Whitney *U* tests were performed with Bonferroni correction for multiple comparisons. The initial uncorrected results suggested differences between breast and lung cancer (*p* = 0.007) and breast and prostate cancer (*p* = 0.019). However, after applying multiple comparison corrections (Bonferroni, Holm and Benjamini‐Hochberg methods), no pairwise comparisons remained statistically significant. Although the Kruskal‐Wallis test detected an overall difference among the groups, the post hoc pairwise tests were not statistically significant after correction, suggesting that the observed effect may be due to slight differences distributed across multiple groups rather than a strong effect between specific pairs. Post hoc analyses using the Mann–Whitney *U* test with Bonferroni correction showed that patients with a net household income of ≤ 600 EUR per person per month faced more significant financial toxicity than those with higher incomes (*p* < 0.001). Additionally, employed patients reported more significant financial toxicity compared to retired individuals (*p* < 0.001). Similarly, religious patients experienced higher financial toxicity than atheists (*p* < 0.001).

Patients with higher household income and those not undergoing active treatment reported better QoL (*p* = 0.007 and *p* < 0.001, respectively). Post hoc analysis showed no significant differences in GHS/QoL across education levels and cancer types. Patients with breast and prostate cancer had slightly better QoL than those with head and neck or other cancers (*p* = 0.018 and *p* = 0.019, respectively), though the differences were not statistically significant after Bonferroni correction. Similarly, patients with incomplete (primary) education had lower GHS/QoL scores than those with secondary education (*p* = 0.024), but this difference was also not significant after correction.

### 3.3 Objective Measures of Financial Toxicity

Objective measures of financial toxicity include direct (i.e., transportation cost, supplements, medical devices, etc.) and indirect costs (i.e., loss of income) related to disease and treatment. Most patients (*n* = 502, 84.8%) reported that their illness and treatment did not incur significant financial expenses.

#### 3.3.1 Direct Costs

##### 3.3.1.1 Transportation

Among direct costs, we explored the type of transportation patients used during active treatment. Most commonly, the patients used their own transportation (*n* = 243, 41.2%) and did not file for reimbursement of travel expenses. The average monthly cost of transportation in this group was 193 €. The second largest group included those who used their own transportation but did file for reimbursement of travel costs (*n* = 136, 23.1%). Patients in this group paid on average 334 € for travel costs, but the latter were reimbursed by health insurance. Additional information on transportation and travel costs is shown in Table 3.

**TABLE 3:** Transportation costs.

Type of transportation	Number	Proportion (%)	Average monthly expenses
Own transport, I don't claim reimbursement of travel expenses	243	41.2%	193 (SD + 296)
Own transport, I claim reimbursement of travel expenses	136	23.1%	334 (SD ± 305)
Public transport or taxi	67	11.4%	193 (SD + 319)
Non‐emergency or other medical transport covered by health insurance	143	24.2%	/

*Note:* Data on transport costs were not available for 1 patient.

##### 3.3.1.2 Out‐of‐Pocket Expenses

**FIGURE 3:**
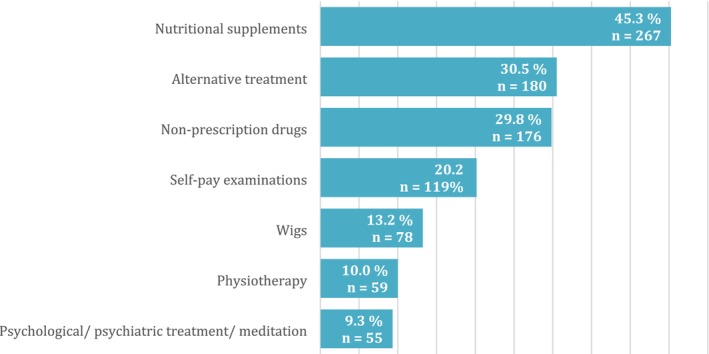
Most common out‐of‐pocket expenses and the proportion of patients who used them.

Patients most often spent out‐of‐pocket on nutritional supplements (*n* = 267, 45.3%), alternative treatment options (*n* = 180, 30.5%) such as bio‐resonance, cannabis preparations and healers and over‐the‐counter medications (*n* = 176, 29.8%). Figure 3 shows additional information on the most common out‐of‐pocket expenses and the proportion of patients using them.

### 3.4 Indirect Costs

A little over half of the patients (*n* = 316, 53.6%) typically visited the Institute alone, while nearly a third (*n* = 173, 29.3%) were accompanied by a companion during their free time. A smaller proportion (*n* = 58, 9.8%) were accompanied by a working companion who used their leave, and the fewest (*n* = 42, 7.1%) were accompanied by a working companion who took sick leave for the visit. One patient did not answer this question. Regarding work absences due to caregiving, nearly a third of patients (*n* = 175, 29.8%) reported that at least one family member took a day off work at least once. A smaller proportion (*n* = 83, 14%) had a family member who took sick leave, while in rare cases (*n* = 11, 1.9%), a family member reduced their working hours due to the patient's illness. Before the onset of the disease, most respondents (*n* = 300, 50.8%) were retired, followed by those employed full‐time or self‐employed (*n* = 244, 41.3%). A small group worked part‐time or were part‐time self‐employed (*n* = 14, 2.4%). Others were unemployed (*n* = 22, 3.7%), received a partial disability pension (*n* = 2, 0.3%), were in education (*n* = 1, 0.2%), or selected another category (*n* = 5, 0.8%). Among those selecting “other,” two were family helpers (The status of family helper is granted to family members of people requiring full‐time care. The government provides partial wage loss compensation to family helpers.), one was on sick leave, one worked under a company contract and one received a widow's pension. Employment data were missing for two patients. At the time of the survey, a little over one‐fifth (*n* = 129, 21.8%) were on sick leave due to the illness specified in the questionnaire. Some patients worked part‐time due to the illness (*n* = 12, 2.0%), while others took partial (*n* = 10, 1.7%) or full (*n* = 18, 3.0%) disability retirement. Some reported losing employment due to the illness (*n* = 9, 1.5%), of whom three (*n* = 3, 0.5%) were actively job‐seeking, while five (*n* = 5, 0.8%) were unable or unwilling to seek work. One individual found new employment in the meantime. Additionally, one patient fell ill during pregnancy and received maternity leave during treatment. Employment data were unavailable for two patients.

## 4 Discussion

In this article, we present the results of the first study in which the COST‐FACIT‐v2 was used for the assessment of financial toxicity in Slovenia. The collected data indicate that the financial burden on cancer patients in Slovenia is low. Most patients (57.2%) did not experience financial toxicity, and 42.8% experienced mild (G1) to moderate (G2) financial toxicity. The causes of financial toxicity are complex. These findings must be interpreted in the context of Slovenia's healthcare coverage model where the treatment and rehabilitation of malignant diseases is fully covered by CHI. As a result, there are no co‐payments for core cancer‐related services. Due to the comprehensiveness of this national insurance system, direct government subsidies or targeted financial assistance were not assessed as separate variables in this study. There is no legal provision for patient‐borne co‐payments for standard oncological care—including surgery, systemic therapy, or radiation therapy—as these services are entirely funded by the public health system. Nonetheless, out‐of‐pocket expenditures may still occur for services not covered by CHI, such as transportation to healthcare facilities, non‐prescription drugs, nutritional supplements and certain complementary or alternative therapies (these categories were captured in Figure 3). Until 2023, patients were required to co‐pay for health services unrelated to their cancer care. However, approximately 95% of the Slovenian population held CoVHI, typically with monthly premiums of 36€, with patients receiving social benefit payments being fully exempt from paying co‐payments. The financial burden due to direct healthcare costs can be considered relatively low. According to Organisation for Economic Co‐operation and Development (OECD) data, Slovenia ranks 22nd among EU countries in terms of out‐of‐pocket payments as a share of total healthcare expenditure, at approximately 12% [33]. It is, however, important to note that direct healthcare expenditures encompass only one element of the overall economic burden of cancer. The latter also includes elements of the burden explored by this paper such as direct non‐medical costs relating to travel costs, food supplements etc. often borne by patients and their families. The economic burden of cancer also reflects productivity losses and costs of informal care that are comparable in size to the direct costs of cancer in Europe [34]. One of the most important factors for financial toxicity is a reduction in income. Patients may experience a loss or reduction in income due to sick leave, part‐time work, or partial or total disability retirement.

The results of our survey confirm statistically significant differences in financial toxicity depending on the level of monthly net income per household (*p* < 0.001). A lower income was associated with a greater financial burden, as confirmed by numerous studies, regardless of the organisation of the health system [19, 35, 36]. In addition, wage compensation during sick leave is regulated by law. It accounts for 80% of an individual's salary for the first 90 calendar days and increases to 90% after 90 days (The first 20 or 21 working days of sick leave are paid by the employer, all subsequent are covered by the compulsory health insurance fund.) [12, 15]. Disability pensioners receive a pension that is usually less than 90% of an individual's salary, depending on the degree of disability. Notably, the financial compensation for sick leave is greater than the disability pension which results in longer periods of sick leave averaging almost a year. Insured persons are entitled to an old‐age pension at the age of 60 if they have 40 years of service or at the age of 65 if they have at least 15 years of insurance. Retired patients do not lose their income because of their illness, unlike employed patients. As a result, older patients may have fewer financial problems than younger patients do and thus a lower level of financial toxicity. These results are consistent with studies from the United States, Europe and Japan [17, 19, 32, 36, 37]. A lower income increases the financial burden and influences a poorer QoL (*p* = 0.007). Patients reported less satisfaction with their personal finances because of the illness. In a study by Honda et al. in Japan, 75.8% of patients with chronic myeloid leukaemia treated with imatinib reported that they experienced financial hardship [38]. In a study by Perrone et al. in Italy, 26% of patients experienced financial hardship due to their disease [21]. Considering the reported results of other studies, the proportion of Slovenian patients struggling with financial burdens is comparable to the average of countries with a well‐developed public health system [19, 37].

There was no statistically significant difference in financial toxicity between men and women. Patients undergoing active treatment (*p* = 0.001) were more susceptible to financial toxicity (*p* = 0.016). Patients who received active oncologic treatment experienced greater financial toxicity and poorer QoL than did those who did not receive active treatment. Similar results were reported in a study of sarcoma patients in Germany [39]. Some studies carried out in the United States and Europe reported higher financial toxicity for women with breast cancer [17, 40, 41, 42] as they tend to be younger and more affected by income loss than patients with other common cancers. Additionally, women earn slightly less than men on average. In our study, the initial results suggested the higher difference between breast and lung cancer but were still not statistically significant. Interestingly, religious people experienced greater financial toxicity and poorer QoL, likely due to an unbalanced sample, as most participants were religious. This was not demonstrated with the QLQ‐C30 FI, where atheists were more prone to financial toxicity (*p* = 0.002). A survey of Latina breast cancer patients revealed a positive link between religion and better QoL [43]. It is assumed that faith helps individuals cope with the disease, symptoms and pain. Some studies have confirmed that patients from rural and remote areas in the USA, Australia and China experience higher financial toxicity. This is primarily due to higher transportation and accommodation costs [2, 44]. In China, an additional factor is outcome inequity caused by the urban–rural dual healthcare system [45]. In Slovenia, we also observed greater financial toxicity in rural areas (*p* = 0.032), likely due to higher transportation costs and lower income levels. However, we must highlight that pensioners in Slovenia have access to free public transportation including buses and trains as of 2020 [46]. The comparison between the COST‐FACIT and EORTC‐QLQ‐C30 revealed a moderate correlation for FI (*ρ* = −0.60, *p* < 0.001) and a mild association for GHS/QoL (*ρ* = 0.38, *p* < 0.001). This suggests that financial toxicity, when assessed using a single question (Q38) in the EORTC QLQ‐C30, may serve as a preliminary indicator for identifying patients at higher risk of financial burden, despite the more comprehensive assessment provided by the 12‐item COST‐FACIT questionnaire. Evaluating the financial burden's impact on QoL is a complex task due to the interplay of economic, psychological and social factors. Unlike straightforward financial metrics, QoL encompasses both tangible and intangible elements, making it difficult to quantify how financial stress directly affects overall well‐being. The perception of QoL is influenced by various factors, highlighting the complex relationship between financial toxicity and QoL, as supported by several studies [17, 21, 47]. What one person considers a financial burden may not be the same for someone else. Emotional and psychological stress caused by financial struggles varies widely among individuals. Although many factors influence QoL, higher financial toxicity in predominantly private healthcare systems is expected to have a greater negative impact on QoL compared to public healthcare systems, where out‐of‐pocket costs are typically lower. Our study revealed that household income and treatment activity were associated with a lower QoL, whereas age, sex, cancer type, education level, religion, living environment, employment status, recurrence and type of treatment had no significant effect.

### 4.1 Advantages and Limitations

The COST‐FACIT questionnaire is a valuable tool for measuring the financial burden of cancer patients. It has the advantage of being short and internationally validated, which allows comparisons between different countries and healthcare systems. It can be used as a screening tool to identify vulnerable patient groups and could be integrated as part of the Patient‐Reported Outcome Measure (COST PROM) [31]. The analysis of financial toxicity with the COST‐FACIT provides a basis on which more complex questionnaires can be added to analyse more complex causes of financial toxicity.

The main strengths of the study are the large sample size and its representation of cancer incidence in the population. However, the uneven distributions of the groups and the nonprobability sample are limitations. For example, some patient groups that are likely to experience greater financial burdens may not have been included (e.g., patients in nursing homes). Some questions were perceived as incomprehensible by our patients. For example, question number one (FT1) is difficult to translate to our setting, as cancer treatment does not depend on patient savings. Furthermore, if two out of twelve questions are missing, the questionnaire becomes invalid, as the analysis cannot be carried out. In this study, only the GHS/QoL was used to compare with the FI scale, simplifying the otherwise complex and multifaceted perception of well‐being. Some studies have also included other EORTC QLQ‐C30 scales (e.g., physical functioning) or other instruments (e.g., Functional Assessment of Cancer Therapy‐General/FACT‐G) that may influence QoL [28, 47, 48]. Objective assessment of financial toxicity is just as crucial as subjective evaluation. Both the COST‐FACIT and EORTC QLQ‐C30 instruments measure only subjective financial toxicity, highlighting the need for additional tools to quantify the actual costs incurred by patients. In public healthcare systems, where cancer treatment is largely covered by insurance, accurately determining the true financial burden on patients remains a challenge.

Certain financial variables were also not assessed separately. For example, financial support from family members was not individually measured; instead, self‐reported net household income per person per month was used, which implicitly accounts for all income sources, including shared household resources. Similarly, housing data were not collected as an independent variable but were indirectly reflected in income levels and living arrangements (e.g., living alone vs. with others).

Despite these limitations, the findings provide valuable insights and highlight areas for further research to enhance the understanding and measurement of financial toxicity in cancer patients.

## 5 Conclusions

The initial results indicate that approximately 40% of cancer patients in Slovenia have mild to moderate financial toxicity. The severity of financial toxicity varies according to monthly income, employment status, age, treatment activity, living in rural areas and religious status. The correlation between financial toxicity and QoL was mild. Using a customized questionnaire to identify patients at higher risk of financial toxicity could enhance patient differentiation and inform more effective health policy.

## Author Contributions

Conceptualisation: Marjeta Skubic, Katja Vöröš, Ivica Ratoša, Helena Barbara Zobec Logar; Methodology: Mojca Bavdaž, Ivica Ratoša, Helena Barbara Zobec Logar; Data curation: Helena Barbara Zobec Logar; Investigation: Marjeta Skubic, Katja Vöröš Andraž Perhavec, Ivica Ratoša, Helena Barbara Zobec Logar; Validation: Marjeta Skubic, Katja Vöröš, Ivica Ratoša, Helena Barbara Zobec Logar; Formal analysis: Marjeta Skubic, Ivica Ratoša, Helena Barbara Zobec Logar; Supervision: Helena Barbara Zobec Logar; Funding acquisition: Ivica Ratoša; Visualisation: Marjeta Skubic, Mojca Bavdaž, Helena Barbara Zobec Logar; Project administration: Ivica Ratoša, Helena Barbara Zobec Logar; Resources: Ivica Ratoša: Writing – original draft: Marjeta Skubic, Helena Barbara Zobec Logar; Writing – review and editing: Marjeta Skubic, Mojca Bavdaž, Petra Došenović Bonča Andraž Perhavec, Tjaša Redek, Ivica Ratoša, Helena Barbara Zobec Logar.

## Conflicts of Interest

The authors declare no conflicts of interest.

## Supporting information


Data S1.

## Data Availability

The data that support the findings of this study are available on request from the corresponding author. The data are not publicly available due to privacy or ethical restrictions.
